# London Mortality Tables for the Quarter Ending January 2

**Published:** 1841-04

**Authors:** 


### PART FOURTH.

### Medical Intelligence.

### TABLE OF MORTALITY FOR LONDON,

### For the Fourth Quarter of 1840:

Showing the number of Deaths from all causes registered in fourteen weeks, from the 26th September, 1840, to the 2d January, 1841.

| Causes of Death. | Total Deaths. | Causes of Death. | Total Deaths. | Cause of Death. | Total Deaths. |
|---|---|---|---|---|---|
| Class i. | | Class iv. | | Class ix. | |
| Smallpox | 738 | Pericarditis | 10 | Ulcer | 7 |
| Measles | 383 | Aneurism | 18 | Fistula | 4 |
| Scarlatina | 465 | Dis. of the heart, &c. | 270 | Diseases of skin, &c. | 5 |
| Hooping-cough | 301 | Total Dis. of Heart, &c. | 298 | Total Dis. of Skin, &c. | 16 |
| Croup | 107 | Class v. | | Class x. | |
| Thrush | 62 | Teething | 245 | Inflammation | 96 |
| Diarrhœa | 87 | Gastritis, enteritis | 221 | Hemorrhage | 44 |
| Dysentery | 19 | Peritonitis | 13 | Dropsy | 522 |
| Cholera | 7 | Tabes mesenterica | 80 | Abscess | 50 |
| Influenza | 22 | Ascites | 11 | Mortification | 62 |
| Typhus | 346 | Ulceration | 23 | Scrofula | 27 |
| Erysipelas | 110 | Hernia | 24 | Carcinoma | 88 |
| Syphilis | 3 | Colic or ileus | 19 | Tumour | 19 |
| Hydrophobia | 0 | Dis. of stomach, &c. | 87 | Gout | 10 |
| Total Epidemic, &c. | 2650 | Hepatitis | 23 | Atrophy | 89 |
| Class ii. | | Jaundice | 23 | Debility | 328 |
| Cephalitis | 159 | Dis. of the liver, &c. | 99 | Malformations | 13 |
| Hydrocephalus | 461 | Total Dis. of Stom. &c. | 848 | Sudden deaths | 226 |
| Apoplexy | 265 | Class vi. | | T. Dis. of Uncert. Seat. | 1574 |
| Paralysis | 243 | Nephritis | 8 | Class xi. | |
| Convulsions | 738 | Diabetes | 7 | Old age or nat. decay | 1047 |
| Epilepsy | 62 | Stone | 2 | Class xii. | |
| Insanity | 12 | Stricture | 5 | Intemperance | 9 |
| Delirium tremens | 23 | Dis. of the kidneys, &c. | 45 | Privation | 10 |
| Dis. of brain, &c. | 106 | Tot. Dis. of Kidneys, &c. | 67 | Violent deaths | 334 |
| Total Dis. of Brain, &c. | 2069 | Class vii. | | Total by Violence, &c. | 353 |
| Class iii. | | Childbed | 119 | Class xiii. | |
| Quinsey | 17 | Ovarian dropsy | 3 | Causes not specified | 64 |
| Bronchitis | 160 | Diseases of uterus, &c. | 47 | | |
| Pleurisy | 27 | Tot. Dis. of Uterus, &c. | 168 | | |
| Pneumonia | 1506 | Class viii. | | | |
| Hydrothorax | 66 | Rheumatism | 39 | | |
| Asthma | 470 | Diseases of joints, &c. | 41 | | |
| Consumption | 1858 | Total Dis. of Joints, &c. | 80 | | |
| Dis. of lungs, &c. | 226 | | | | |
| Total Dis. of Lungs, &c. | 4330 | | | T. Deaths from all causes Males 6901, Females 6655 | 13556 |

